# 1429. Epidemiology, Clinical Characteristics, and Outcomes of Extra-Pulmonary Non-Tuberculous Mycobacterial Infections from a Single Center over a 10-Year Period

**DOI:** 10.1093/ofid/ofac492.1258

**Published:** 2022-12-15

**Authors:** Mary B Ford, Jason Okulicz, Jesse Salinas, John L Kiley

**Affiliations:** SAUSHEC, San Antonio, Texas; Brooke Army Medical Center Infectious Disease Service, Infectious Disease Clinical Research Program, San Antonio, Texas; BAMC, San Antonio, Texas; BAMC, San Antonio, Texas

## Abstract

**Background:**

Non-tuberculous mycobacteria (NTM) cause a wide variety of clinical syndromes, including extra-pulmonary infections. Skin and soft tissue infections (SSTI) and bone infections caused by NTM are often associated with traumatic inoculation and data to guide diagnosis and treatment is limited. We sought to better understand skin and soft tissue infections (SSTI) and bone infections caused by NTM.

**Methods:**

All NTM clinical isolates recovered at Brooke Army Medical Center from 2012-2022 were initially screened and SSTI and bone isolates were included. Corresponding electronic health records were reviewed for epidemiologic, microbiologic, and clinical data. Infections were defined as recovery of ≥ 1 NTM isolate from skin, soft tissue, or bone cultures with a corresponding clinical syndrome.

**Results:**

Forty isolates from 27 corresponding patients were analyzed with a median of 1 isolate [IQR 1-1] per patient. Two-thirds (18, N = 27) were female with a median age of 51 years (IQR 33-66, Table 1). A total of 21 (78%) patients had infecting isolates, most commonly secondary to surgery (28.6%) or trauma (33%). Six of 21 (29%) had bone infections, 5 (83%, N = 6) were secondary to trauma, and 2 (33%, N=6) had both bone and SSTI isolates recovered. The majority were rapid growers (81%), most frequently *M. abscessus*. In infected patients, 8 (38%) had combined medical/surgical therapy, 8 (38%) had surgery alone, 4 (19%) had medical therapy alone, and 1 (5%) patient received no treatment. No infectious disease consultation was sought for 7 (87.5%) patients who received only surgery nor for the patient who received no treatment. Time from symptom onset to isolate recovery was a median of 64 days (IQR 48-108), with a median of 33 days (IQR 6-60) from isolate recovery to directed medical therapy. At the end of the study period 2 of 21 patients had died.

**Figure:**
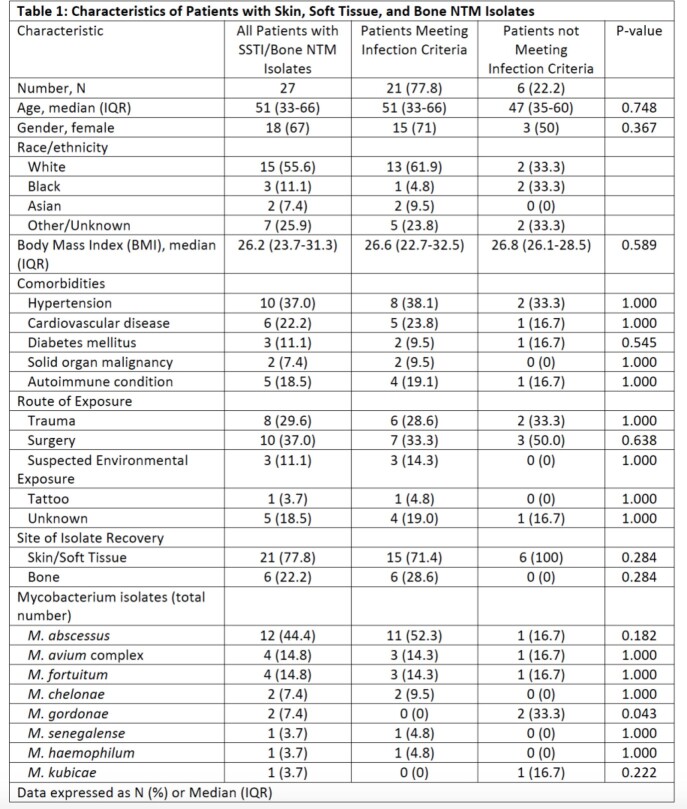

**Conclusion:**

Data supporting diagnosis and treatment decisions in SSTI and bone NTM infections is sparse. In this study the majority of NTM isolated were determined to be the cause of true infections. We confirm that surgery and trauma are the most common mechanisms of exposure. The delay between symptom onset and directed therapy demonstrates a need for additional studies delineating specific criteria for diagnosis and treatment.

**Disclosures:**

**All Authors**: No reported disclosures.

